# Compensation Strategies in Post-Stroke Individuals: Insights from Upper Body Kinematics Analysis Based on Inertial Sensors

**DOI:** 10.3390/s25247609

**Published:** 2025-12-15

**Authors:** Carrie-Louise Thouant, Elena Sofia Cocco, Giovanni Morone, Carlotta Maria Manzia, Francesco Infarinato, Paola Romano, Matteo Cioeta, Michela Goffredo, Marco Franceschini, Sanaz Pournajaf

**Affiliations:** 1 Neuromotor Rehabilitation and Rehabilitation Robotics, IRCCS San Raffaele Roma, 00163 Rome, Italy; carrielouise.thouant@sanraffaele.it (C.-L.T.);; 2 San Raffaele Institute of Sulmona, Viale dell’Agricoltura, 67039 Sulmona, Italy; 3 Department of Life, Health and Environmental Sciences, University of L’Aquila, 67100 Aquila, Italy; 4 Rehabilitation Bioengineering Laboratory, IRCCS San Raffaele Roma, 00163 Rome, Italy; 5 Department of Medicine and Health Science “Vincenzo Tiberio”, University of Molise, 86100 Campobasso, Italy; 6 Department of Human Sciences and Promotion of the Quality of Life, San Raffaele University, 00166 Rome, Italy

**Keywords:** stroke, upper limb, inertial measurement units, compensation strategies

## Abstract

**Highlights:**

**What are the main findings?**

- The use of inertial measurement units (IMUs) during the Box and Block Test enabled detailed kinematic analysis and the identification of typical compensation strategies in post-stroke individuals.
- Overuse of the wrist, shoulder, and trunk was quantified, with 88% of participants showing compensation at the wrist and trunk, and 68% at the shoulder.

**What is the implication of the main finding?**

- IMUs provide a simple, eco-friendly, and effective tool for objectively assessing movement quality in clinical settings.
- Detecting and quantifying compensation supports the development of personalized rehabilitation approaches and contributes to optimizing functional recovery after stroke.

**Abstract:**

Background: One of the main goals of rehabilitation after stroke is the restoration of motor function. Understanding movement patterns and compensatory strategies is essential to optimize therapy. This study analyzes upper limb kinematics during the Box and Block Test (BBT) to identify and quantify typical post-stroke compensation strategies. Methods: Thirty-one sub-acute stroke participants and thirty-one healthy controls were included. Kinematic data were collected using a 7-IMU system. Joint angles were analyzed with MATLAB R2023a, and 3D trajectories were reconstructed from calibrated quaternions and anthropometric data. Group differences were assessed with the Mann–Whitney test. Compensation strategies were quantified in percentage terms relative to healthy subjects. Results: Significant intergroup differences were observed in mean joint angles and ranges of motion. On the paretic side, participants overused the wrist and shoulder to compensate for reduced elbow and trunk activity. Similar overuse was also observed on the unaffected side. Quantification showed that 83.9% and 80.6% compensate, respectively, with wrist and trunk and 67.7% with the shoulder. Conclusions: Using IMUs during the BBT, this study identified specific compensation strategies that may hinder recovery. It also contributed to developing a quantification scale, supporting more personalized rehabilitation and improved quality of life.

## 1. Introduction

In the field of post-stroke upper limb rehabilitation, restoring motor function remains a key objective [1]. To optimize therapeutic interventions and promote effective recovery, it is essential to objectively understand the movement patterns and compensatory strategies adopted by individuals after a stroke, as these aspects have a direct impact on their autonomy in daily [2]. Stroke, identified as a leading cause of adult disability globally [3,4], frequently leaves individuals with debilitating upper limb motor impairments [5], necessitating the development of alternative strategies to perform daily activities. Following upper limb impairments, compensatory movements may be observed, where unaffected muscles, joints, and effector body segments are utilized and controlled by alternative brain regions to accomplish the required tasks and achieve goals [6]. While these compensatory strategies may initially aid in mitigating motor deficits, their long-term utilization can compromise movement quality, leading to chronic issues such as pain, reduced muscle capacities, and diminished motor recovery efficacy that can interfere with the physiological movement pattern [7,8,9,10]. Therefore, understanding and objectively characterizing compensatory movements is essential to guide effective rehabilitation and support functional independence after stroke [11,12,13,14].

The Box and Block Test (BBT), a widely employed measure for assessing manual dexterity in post-stroke persons, typically provides a global score without offering a detailed evaluation of movement quality [15]. However, it is quick, simple, and cost-effective and thus appropriate for use in the routine clinical setting [16] to characterize the effectiveness of a specific therapy or disease progression over time [17,18,19].

In this context, kinematic analysis during BBT execution could allow for an accurate and objective assessment of upper limb and trunk movements, providing a unique opportunity to identify and comprehend the compensatory strategies employed by post-stroke individuals [20,21]. Indeed, recent research by Wang et al. [22] demonstrates the importance of considering compensatory strategies in post-stroke rehabilitation. The literature concerning the kinematic studies of the upper limbs during clinical tests is abundant but diverse in terms of analysis technology and the types of activities studied [23,24,25,26,27,28,29,30,31]. The use of Inertial Measurement Units (IMUs) as an alternative to stereophotogrammetry has made motion analysis feasible in both clinical settings and ecological environments. However, despite this growing interest, no study to date has quantified compensatory strategies during the Box and Block Test in post-stroke individuals using a validated inertial system. This gap limits the possibility of integrating objective compensation assessment into clinical practice.

Recently, research by Romano et al. [23] examined upper body movements while performing a modified variant of BBT using IMU technology, both in healthy people and those diagnosed with Parkinson’s disease (PD) suggesting adaptation techniques used by people with PD.

These results suggest that an IMU-based analysis system could be integrated into post-stroke rehabilitation setting to facilitate precise and objective evaluations of movement patterns, thereby offering an unparalleled opportunity to examine compensatory strategies during BBT [23].

To our knowledge, this is the first study to provide a detailed quantification of wrist, shoulder, and trunk compensations during the BBT using a validated 7-IMU system in both post-stroke individuals and healthy controls. This novel approach goes beyond existing studies by offering an objective, scalable, and clinically interpretable compensation measure.

Furthermore, to improve clinical interpretation, we introduce a compensation scale on levels from 0 to 10 derived from the quantified differences between post-stroke participants and healthy subjects in terms of mean joint angle for each joint segment calculated during the BBT performance. This scale provides an objective grading of upper limb compensations, offering clinicians an intuitive tool to assess the severity of compensatory movements and to monitor their evolution over time.

The aim of this study is to investigate upper body kinematics and compensatory strategies using an IMU-based system, during the BBT execution in individuals with stroke compared to healthy persons. Our hypothesis is that an IMU-based system might provide an accurate description of movement quality and could enable the quantification of typical compensatory strategies.

## 2. Materials and Methods

### 2.1. Study Design

This observational study took place within the Neuromotor Rehabilitation and Robotic Rehabilitation Research Area and Rehabilitation Bioengineering Laboratory at IRCCS San Raffaele Roma (Rome, Italy). The study adhered to the principles outlined in the Declaration of Helsinki and received approval from the regional ethics committee “Comitato Etico IRCCS San Raffaele, Pisana” (no. PR. 19/34, dated January 2020). All health and safety procedures were followed during the study.

### 2.2. Participants

Subjects who met the inclusion criteria admitted to the IRCCS San Raffaele from February 2020 to October 2023 were assessed for suitability for the study. All individuals recruited for the study signed informed consent documents to participate in the research.

#### 2.2.1. Control Group

Adults aged 50 to 80 years, with no upper limb pathology, cognitive impairment, or severe visual deficit preventing the execution of the BBT motor task, were included in the Control Group (CG). All participants in the CG must have a dominant right limb.

#### 2.2.2. Stroke Group

Individuals in the early subacute phase of stroke [32] were included in the Stroke Group (SG) if they met the following inclusion criteria: diagnosis of a first stroke; aged between 50 and 80 years; ability to maintain a seated position on a chair without support for at least 30 min [33]; moderate or mild hemiparesis affecting upper limb motor performance due to the condition, measured using the Fugl-Meyer Assessment Upper Limb (FMA-UL) (22 < FMA-UL ≤ 44); and a Modified Ashworth Scale (MAS) score of the main components of the upper limb < 3. We did not include individuals with unstable general medical conditions, an inability to understand study instructions, cognitive disorders, spatial neglect, recent Botox injections in the upper limb, psychological disturbances interfering with equipment use or testing, or pre-existing joint pathologies prior to the stroke that limit the range of motion of the upper limbs. According to the inclusion criteria, the location of the cortical or subcortical lesion must correspond to Bamford’s classification: total anterior circulation infarct (TACI) and partial anterior circulation infarct (PACI). Conversely, according to the exclusion criteria, lesion locations corresponding to posterior circulation infarct (POCI) and lacunar infarct (LACI) are excluded.

### 2.3. Clinical Assessments

The evaluation of tone, strength and motor skills was assessed by the total FMA-UL scores, the trunk stability by TCT, the spasticity by MAS, the execution velocity with the standard BBT [17]. Clinicians with expertise in movement disorders, trained in administering and interpreting the tests, assessed each one. The standardized tests and BBT acquisition with IMUs were performed consecutively during the same session, lasting one hour. To determine the laterality and therefore the dominance of one hand over the other, participants were asked to respond to the 10-item version of the Edinburgh inventory as suggested by the article of Sainburg and Kalakanis [34].

### 2.4. Experimental Setup–IMU Setup & Calibration

**Figure 1:**
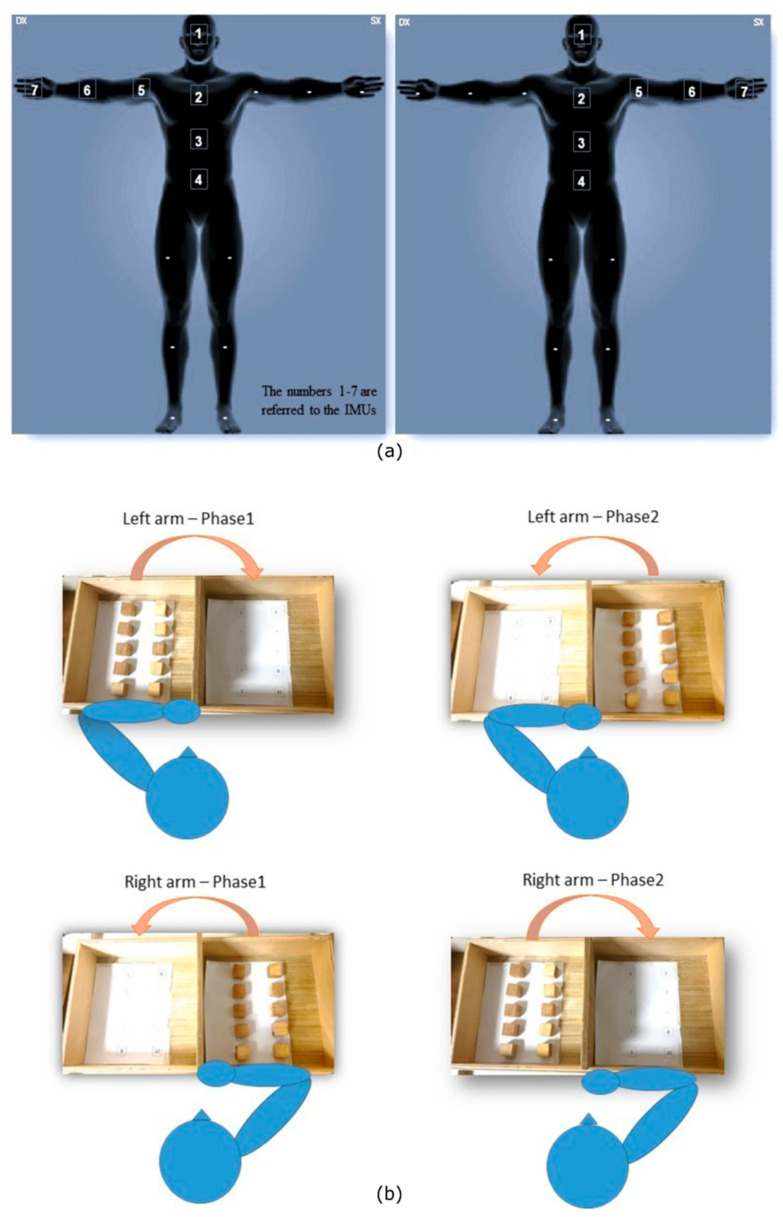
(**a**) Placement of the inertial measurement units (IMUs) on the body: head (sensor 1), trunk (sensors 2–3–4), and upper limb (sensors 5–6–7). (**b**) Experimental setup of the IMU-based targeted Box and Block Test, showing the subject’s positioning and the arrangement of the task components. The setup highlights the distinction between Phase 1 and Phase 2, which are analyzed separately throughout the study.

The study was conducted at the Research Area in Neuromotor Rehabilitation and Robotic Rehabilitation (IRCCS San Raffaele, Roma), equipped with the MOVIT IMU sensor network (Captiks s.r.l., Rome, Italy). The MOVIT system was validated using a video-based reference system [35] (Vicon, from Oxford Metrics) and the results demonstrated excellent accuracy and repeatability (RMSE errors joint angles < 3.5°, the testing was realized by measuring different subjects performing different motor exercises). The study used 7-IMU positioned to reconstruct trunk–arm kinematics according to established biomechanical models. Sensors were placed on head, seventh cervical vertebra–C7, tenth thoracic vertebra–T10, fifth lumbar vertebra–L5, Mid-arm, mid-forearm, dorsum of the hand at the III metacarpal. Placement followed the recommendations of Saggio et al. (2021) [35] and Vitali & Perkins (2020) [36] for anatomical frame identification and minimization of soft-tissue artifacts. Each sensor was securely fastened with Velcro elastic straps to reduce micro-movements relative to the skin. The experimental setup and the positioning of the IMUs on the arm are shown in Figure 1. The chosen configuration represents the minimum number of sensors needed to reconstruct trunk and upper limb kinematics relevant to compensatory strategies, estimate 3D hand trajectories and fluidity metrics, and, finally, maintain a simple, environmentally friendly, and clinically feasible instrumentation. To optimize accuracy and minimize drift, a two-stage calibration procedure was used.

- First calibration phase: 90° rotations around the three axes (to ensure a global reference and define the initial alignment of the quaternion reference system).
- Second calibration phase–Functional Alignment (FA): participants, after consistently positioning the sensors (Figure 1), performed a standardized T-pose [37]. This second stage is necessary to align the sensor axes with the anatomical axes and reduces systematic offsets in joint angle estimation.

Data were recorded at a sampling rate of 60 Hz, appropriate for capturing upper limb movements in clinical functional testing. The chosen frequency is consistent with the typical bandwidth of human upper limb movement and with previous validation studies of the IMU in post-stroke and Parkinson’s disease populations [23,38].

### 2.5. Task Description

At the beginning of the test, the participant adopts a neutral posture: a straight back, relaxed shoulders without elevation, head aligned with the spine, and feet flat on the floor with knees bent at approximately 90°. The forearms rest on the table, forming a 90° angle with the upper arms, while the hands, relaxed, are placed flat on the surface. The elbows remain close to the body. Participants wore appropriate clothing to ensure proper adhesion of the Velcro straps, thereby guaranteeing optimal sensor fixation.

The subjects performing were divided in two phases for each arm: (i) Phase 1, during which subjects are requested to transfer the blocks from the ipsilateral side with respect to the moving arm to the other one and (ii) Phase 2 for the opposite displacement. After the measurement of anthropometric data (C7-T10 distance, T10-L5 distance, upper arm and forearm length), participants performed the requested motor task starting with the dominant arm and then with the non-dominant arm, regardless of the location of the lesion.

### 2.6. Data Processing

Data acquisition was carried out using the Captiks IMU system (Captiks s.r.l., Rome, Italy), which includes Motion Studio for system configuration and recording and Motion Analyzer for extracting joint-angle curves and calibrated quaternions in .csv format. The exported data were subsequently processed using an in-house MATLAB algorithm (MATLAB R2023a, The MathWorks, Natick, MA, USA), specifically developed for this study.

The workflow consisted of multiple steps. The raw data from the IMUs were imported using a dedicated function, subsequently filtered and segmented. The data were segmented into ten trials, in which the beginning of the single trial corresponds to the moment the block is grasped, and the end corresponds to the moment the same block is released. The task was segmented into ten trials, corresponding to the transfer of the ten standardized wooden cubes placed within the 5 × 2 grid of the box, which was designed to replicate the spatial layout and proportions of the standard BBT. For each participant, the ten block transfer cycles were first analyzed individually and then calculated as a subject-level average, allowing for comparisons between individuals and the subsequent generation of group-level mean curves. Joint angles were calculated on normalized time bases (0–100% trial completion) to allow for comparisons between subjects.

Finally, using calibrated quaternions, rotation matrices, and anthropometric measures (C7-T10 distance, T10-L5 distance, upper arm, and forearm length), the in-house MATLAB algorithm reconstructed 3D hand trajectories through the transformation matrices (1). The goal was to determine body segment orientation using calibrated quaternion coefficients and rotation matrices (R) derived from anthropometric data and sensor distances $\vec{v}$ (the transformation matrices between two consecutive coordinated systems of proximal and distal segment couples is described by the Formula (1)). The pose of the hand was obtained by concatenating the transformation matrices connecting distal and proximal segments.

(1) Tdistalproximal=Rproximal∗Rdistalv→0 0 01

Mean joint angles, Range Of Execution (ROE), mean velocity $(V_{m}$), and fluidity metrics (Dimensionless Jerk, DLJ (2); and Logarithmic Dimensionless Jerk, LDLJ (3)) were calculated. These parameters were extracted for each segmented trial and averaged per subject.

The DLJ and the LDLJ, defined below, are valid jerk-based measures of movement smoothness [38,39,40]: a higher value indicates less fluid and jerky movement. A lower value indicates more fluid and controlled movement. LDLJ is the logarithmic version of DLJ, used to make the values easier to interpret.

(2) $$DLJ=-\frac{{\left(t_{2}-t_{1}\right)}^{5}}{v_{peak}^{2}}\int_{t_{1}}^{t_{2}}{\left|\frac{d^{2}v\left(t\right)}{{dt}^{2}}\right|}^{2}dt$$

(3) $$LDLJ=-ln\left|DLJ\right|$$

where $t_{1}$ and $t_{2}$ are the instants of gesture start and end, respectively, v(t) is the movement speed and $v_{peak}$ is its maximum in the interval [$t_{1},t_{2}$].

### 2.7. Compensation Levels Calculation

To quantify the shoulder, wrist and trunk compensations that were highlighted starting from the results on the comparison between healthy and neurological subjects, the statistically significant differences were calculated in terms of mean angle of shoulder flexion-extension, wrist flexion-extension and lateral bending of the trunk. These differences were calculated in percentage terms with respect to the standard mean angle value obtained for those same joint angles from the CG and subsequently the min-max normalization method was applied, i.e., a linear mapping to transform the percentage values into the desired range. In this case, we mapped the 0 percentage to 0 on the scale and the maximum percentage (100%) to 10 on the scale. The other percentage values were interpolated proportionally between these two extremes. This normalization procedure was adopted to provide an intuitive, clinician-friendly scale for visualizing compensation magnitude. Mapping the 0–100% percentage difference onto a 0–10 range allowed a compact representation consistent with other clinical rating scales. The analyzed quantities were obtained starting from the results of the affected limb of neurological subjects for both phases. This normalization approach was also selected to align with clinical reasoning needs: providing a 0–10 scale enables rapid interpretation by clinicians and parallels the structure of commonly used clinical scoring tools. Furthermore, this methodological design responds to a gap identified in the literature. As emphasized by Wang et al. [22], existing assessments of upper-limb activity rarely quantify compensations in an objective manner, and technology-based solutions remain heterogeneous and not standardized. By deriving the scale directly from the quantified kinematic deviations between post-stroke participants and healthy controls, we provide an initial standardized framework for describing compensation severity during a functional task.

This compensation scale should be viewed as an exploratory quantitative index based on kinematic differences between groups, designed to provide an initial objective framework for describing compensation levels and to support future clinical validation. Future studies will be needed to validate this scale through test–retest reliability, inter-rater agreement, and correlations with other potential compensatory measures.

### 2.8. Statistical Analysis

The sample size calculation was carried out with G*Power 3.1.9.7. A *t*-test was carried out and the following parameters were set power equal to 0.85, type I error equal to 0.05, and effect size equal to 0.8. The total sample size calculated is 60 subjects (i.e., 30 subjects per group). All computed parameters were averaged per subject across blocks before performing statistical analysis. Due to the non-normal distribution of the data (Shapiro–Wilk test) and the number of groups equal to two the non-parametric Mann–Whitney U test was employed to compare each parameter between the CG and the SG, with alpha set at 0.05 (IBM SPSS Statistics for Windows, Version 26.0. Armonk, NY, USA: IBM Corp). It has been demonstrated that differences appear in upper limb motor patterns between dominant and non-dominant limbs, and post-stroke subjects tend to use the unaffected limb (ipsilesional limb) as dominant limb [38,39,40,41]. The values obtained from the unaffected limb (SG) are compared with those obtained from the dominant limb (CG) while the values obtained from the affected limb (contralesional limb, SG) were compared with those obtained from the non-dominant limb (CG) [38,39,40,41]. The statistically significant differences in terms of joint angles for each joint segment allowed us to develop the previously mentioned compensation scale in a second analysis.

Finally, the standard BBT between groups was compared with the Wilcoxon rank-sum test setting alpha equal to 0.05.

## 3. Results

Thirty-one individuals with stroke (SG) and thirty-one healthy individuals (CG) were enrolled in the study. Table 1 outlines the clinical and demographic characteristics of the participants and presents the statistical test results for the pairwise comparisons mentioned earlier. The only clinical test that performed both the CG and SG was the standard BBT, which showed a statistically significant difference between the two groups in terms of the number of blocks moved from one side of the box to the other within one minute for both limbs tested.

The data analysis calculated the joint angle parameters resulting from kinematic acquisition during Phase 1 and Phase 2 for Dominant Arm (DA)/Non-Dominant Arm (NDA) and Unaffected Arm (UA)/Affected Arm (AA). In Table 2, the kinematic parameters derived from the hand trajectories are presented. Statistically significant differences in all parameters between the two groups are demonstrated. Indeed, the average speed and acceleration are significantly lower in the SG than in the CG. Additionally, the DJL index indicates that the neurological subjects had a lower smoothness of movement compared to the healthy subjects, with the trajectories showing more pronounced fluctuations along the movement path.

The mean joint angles registered significant differences between the SG and the CG during Phase 1 (Table 3 and Table 4) in the elbow, pelvis, shoulder, trunk, and wrist joint angles. The Range Of Execution (ROE) index exhibits statistically significant differences during Phase 1 in the elbow, pelvis, shoulder, trunk and wrist joint angles. Specifically, Table 3 shows that these differences involve the elbow, shoulder, and wrist, both in terms of mean angle and ROE. Table 4 further indicates that additional significant differences emerged at the trunk level, particularly in lateral bending, rotation, and pelvic tilt, confirming that trunk and pelvic also contributed to the altered movement strategy in the SG. Based on the quantitative and objective analysis, the increased shoulder ab/adduction and rotation, the reduced involvement of the elbow, and the greater lateral flexion of the trunk and pelvis collectively reflect a compensatory strategy used by post-stroke individuals to achieve the upper-limb motor task.

The mean joint angles and ROE registered significant differences between the SG and the CG during Phase 2 (Table 5 and Table 6). Similarly, during Phase 2 (the return phase of the same motor task) the same joint kinematic patterns emerged, with comparable alterations in upper-limb (Table 5), trunk, and pelvic movements (Table 6) as those observed in Phase 1 (Table 3 and Table 4).

**Figure 2:**
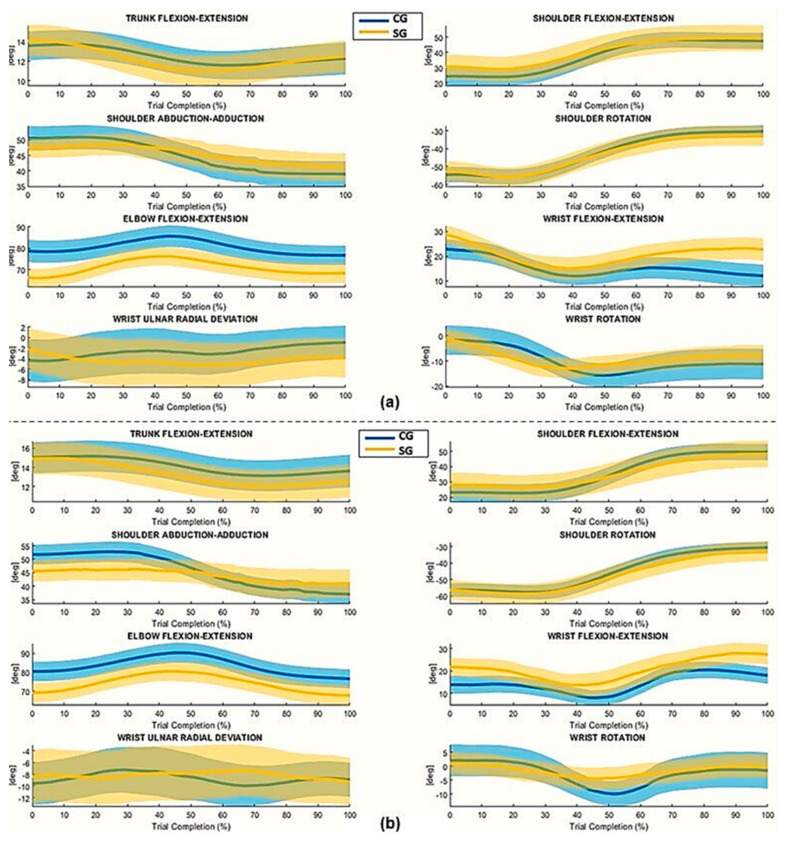
Time-normalized joint angles of the Dominant/Unaffected Arm (DA/UA) during (**a**) Phase 1 and (**b**) Phase 2. The solid line represents the mean trajectory across blocks and participants, plotted over normalized movement duration (0–100%). The shaded area represents the corresponding standard error (± SE). Joint angles are expressed in degrees (°).

**Figure 3:**
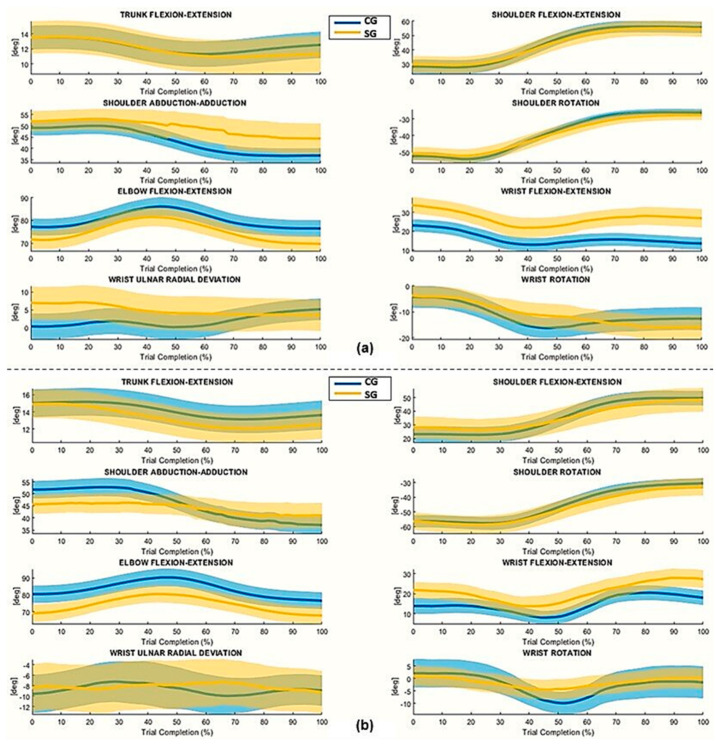
Time-normalized joint angles of the Non-Dominant/Affected Arm (NDA/AA) during (**a**) Phase 1 and (**b**) Phase 2. The highlighted mean line indicates the averaged trajectory across subjects and blocks, while the shaded region depicts the standard error (± SE). Joint angles are reported in degrees (°) over normalized time (0–100%).

Figure 2 and Figure 3 show mean joint angle trajectories over the trial complexion % for both phases. The study of the mean articulation angle trajectories showed that the proposed protocol highlights the different motor strategies used during the execution of the movement. Figure 2 and Figure 3 show the mean joint angle trajectories during the normalized duration of movement for both phases. In Phase 1, the SG exhibits reduced elbow flexion-extension excursions and a flatter pronation-supination profile compared to the CG, while the shoulder exhibits greater abduction/adduction, flexion-extension, and rotation movements. Wrist kinematics also show greater variability and greater angular excursions in the SG, particularly in flexion-extension and ulnar-radial deviation. In contrast, the CG trajectories appear more compact, stable, and symmetrical between the dominant and nondominant sides, indicating more efficient and consistent movement execution. Similarly, trunk trajectories show clear differences between groups. The SG exhibits greater trunk flexion, especially during the blocks corresponding to the initiation of the movement and the transport of the block, while the CG maintains more stable trunk involvement. These results indicate that participants with stroke relied more heavily on the proximal and trunk segments to support task execution. Together, the trajectory profiles in Figure 2 and Figure 3 confirm that the proposed protocol effectively distinguishes the different motor strategies adopted by the two groups during movement.

**Figure 4:**
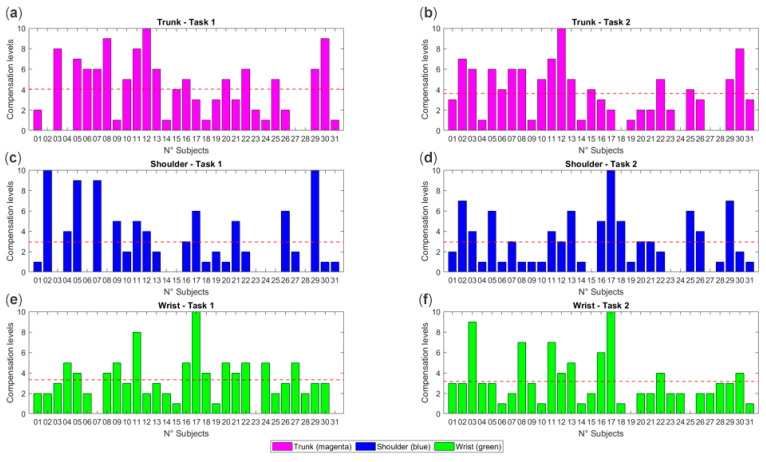
Compensation levels for each subject based on the 0–10 scoring system defined in the Methods (0 = no visible compensation; 10 = maximum compensation according to the predefined observational criteria). Panels report compensations for the trunk ((**a**) Phase 1; (**b**) Phase 2), shoulder ((**c**) Phase 1; (**d**) Phase 2), and wrist ((**e**) Phase 1; (**f**) Phase 2). Each bar represents the subject-specific compensation rating; the x-axis reports subject IDs and the y-axis the compensation score. Colors are used consistently across panels to differentiate the anatomical districts: magenta for trunk, blue for shoulder, and green for wrist, as indicated in the legend.

Figure 4 illustrates the distribution of compensatory behaviors in the stroke group using a 0–10 scale for wrist, shoulder, and trunk compensations. As shown, most participants exhibit higher compensation levels at the trunk and shoulder, a result that is consistent with the kinematic findings reported in the mean joint angle and ROE analyses (Table 3, Table 4, Table 5 and Table 6). However, the figure also highlights a marked inter-subject variability, with some individuals displaying different combinations of compensatory patterns across the three anatomical districts. This approach led to the development of subject-specific graphs quantifying compensation levels (0–10) for the wrist, shoulder, and trunk across both phases of the task. Considering scores from 1 to 10 as indicative of a compensatory pattern, 83.9% of the stroke participants showed consistent wrist compensation in both phases, 80.6% exhibited trunk compensation, and 67.7% presented shoulder compensation. Most individuals displayed multidistrict compensations, with nearly half of the sample showing concurrent compensations at the wrist, shoulder, and trunk, whereas only a small subset of subjects exhibited compensation predominantly confined to a single anatomical district. These findings illustrate the heterogeneous compensatory strategies adopted by post-stroke individuals during task execution. The scale used in Figure 4 therefore provides an objective means to capture these differences, enabling the identification of individual compensation profiles that can support personalized clinical assessment and tailored rehabilitation strategies.

## 4. Discussion

This study aimed to provide the upper limb joint kinematics and identify and quantify compensatory movements in individuals affected by stroke. The results obtained from the healthy subjects were consistent with the literature [20]. The analysis of stroke participants enabled a detailed characterization of movement quality and the identification of specific compensation strategies.

### 4.1. The Affected Limb

Regarding the affected limb in post-stoke persons, trunk rotation, as well as wrist flexion/extension, exhibited a significantly greater range of motion compared to movements in healthy subjects. Additionally, elbow flexion/extension was significantly lower compared to the CG, observed during both the assigned phases. These findings suggest that reduced elbow mobility leads individuals to recruit proximal (trunk) and distal (wrist) segments to complete the task, consistent with well-documented compensatory patterns. These adaptations are thus referred to as compensation strategies, a notion also supported by existing literature [22,38,42,43]. Shoulder abduction was also significantly increased in the SG, reflecting an altered movement strategy throughout both phases, further supporting the presence of compensatory behavior. These results align with previous reports describing how individuals post-stroke reorganize movement toward less efficient but achievable motor solutions [38].

### 4.2. The Unaffected Limb

Regarding the unaffected limb, joint amplitudes were significantly higher across the wrist, shoulder, elbow, and trunk compared to CG indicating overuse of the unaffected limb. Although less frequently reported, this overuse phenomenon has important neurophysiological implications [44]. Furthermore, a quantitative analysis of hand trajectories (Table 2) reveals a significantly lower average speed in the unaffected hand compared to both hands of healthy subjects during both phases. Additionally, the unaffected limb of stroke subjects retrieves a minimal number of cubes compared to healthy subjects for both limbs. This also confirms the impact of the stroke on the non-paretic side of the subjects. A more in-depth analysis of this observation would be highly informative. A plausible explanation is that increased reliance on the unaffected limb becomes a reinforced behavior: repeated use strengthens its motor pathways while simultaneously reducing opportunities for the affected limb to engage in functional tasks. However, it is worth noting that the existing literature on the topic highlights that the tendency to overuse the unaffected limb exacerbates the issue by limiting the use of the affected side, thereby impeding functional recovery [22,42,43,44]. The study conducted by Jones et al. [7] revealed that the acquisition of new strategies for using the unaffected limb leads to neural modifications that interfere with the favorable cerebral changes aimed at enhancing the affected side. This observation could shed light on the persistence of neglecting the affected side, even among individuals with mild deficits. This hypothesis is in line with studies conducted by Taub and co-workers that conceptualized the “learned non-use” principle [45].

### 4.3. Compensation Levels

The kinematic analysis has also allowed quantifying compensation strategies based on normative results from healthy subjects (Figure 4). This approach led to the development of graphs showing the level of compensation for the wrist, shoulder, and trunk in each SG subject. These findings confirm that compensations are not occasional but represent a dominant motor strategy in most individuals post-stroke. The analysis of the compensatory scores further confirms the heterogeneous nature of post-stroke motor strategies. Although most individuals showed compensation at the wrist and trunk (83.9% and 80.6%, respectively) and a smaller proportion at the shoulder (67.7%), the distribution of these behaviors was highly variable across subjects. Nearly half of the participants exhibited combined compensations across all three anatomical districts, suggesting that stroke-related motor impairments often lead to multidistrict adaptations rather than isolated joint compensations. This variability highlights the individualized ways in which post-stroke individuals reorganize movement to accomplish the task. Importantly, the compensation scale used in this study provided an objective means to quantify these distinct profiles, offering clinically meaningful information that can support personalized rehabilitation planning and the development of targeted interventions aimed at reducing maladaptive compensatory behaviors.

It is important to note that there is still a lack of scientific work on this topic [42,46,47] explaining that the focus often lies on the study itself and less on the direct impact of the research in clinical practice. Indeed, Wang et al. [17,22] recently advocated for the continuous development of standardized compensation norms to create a usable standardized assessment in a clinical context. By performing the BBT with the IMUs during his hospitalization, the person allows the therapist to access the scale of compensation levels. This one allows us, firstly, to evaluate the severity of compensatory strategies and, secondly, to follow the potential improvements of his condition over time. This information guides the therapist in the optimization of the treatment and facilitates the implementation of truly personalized support.

### 4.4. Clinical Implications

This study proved to be a feasible, simple, cost-effective, and eco-friendly system. All recruited participants completed motor tasks without encountering difficulty. The protocol included post-stroke individuals and healthy subjects, allowing for the analysis and quantification of typical movements related to stroke.

In an era where precise assessment of motor performance becomes increasingly essential for individualized motor rehabilitation, this system provides the means to conduct an objective and quantitative analysis of movement, thus strengthening the ability of clinicians to detect functional deficits early and adjust their interventions in a targeted manner. Moreover, it facilitates transmission within the clinical context for the benefit of healthcare professionals. Additionally, this approach could introduce the future development of a standardized and validated compensation levels scale, allowing visualization of individual-specific compensations. Analyzing this scale could contribute to a better functional understanding of post-stroke individuals, visualizing their evolution over time and guiding practitioners in therapeutic planning, prioritization of objectives and longitudinal monitoring of progress.

These translational aspects of this research highlight the additional strength of our discoveries since the clinical routine can benefit from its results, by integrating a simple and reproducible tool directly usable in the clinic. This system thus offers concrete support for therapeutic decision-making, improves the accuracy of assessments and potentially contributes to optimizing care pathways in rehabilitation.

### 4.5. Limitations

The present study involved participants with stroke presenting different impairment severities and lesion locations (TACI and PACI), which reflects the variability typically encountered in clinical practice. Nonetheless, this heterogeneity may have contributed to the range of movement strategies observed. Future studies with stratified subgroups based on impairment level and lesion type may allow a more detailed characterization of specific compensation patterns in individuals affected by a stroke. In addition, lesion-site information was not analyzed separately in the current work. Although the IMU system employed has been previously validated, the study did not include a direct comparison with an optical motion capture system. Integrating both technologies within the same protocol in future research could further support methodological robustness. As with all IMU-based methods, the system is subject to potential limitations such as sensor noise, soft-tissue artifacts, and gradual orientation drift. These aspects were mitigated through multi-step calibration procedures, filtering, and quaternion-based processing; however, small residual errors cannot be completely excluded. Future studies may include dedicated analyses of calibration drift, as well as direct comparisons with optical motion capture, to further characterize measurement accuracy. Finally, the compensation scale introduced in this study must be considered an exploratory tool rather than a validated clinical instrument. Although it provides an objective, standardized, and clinically interpretable quantification of compensatory movements, it has not yet undergone formal psychometric validation. Future work is required to assess its test–retest reliability, inter-rater agreement, sensitivity to change, and convergent validity before it can be used as a clinically validated scale.

## 5. Conclusions

In conclusion, this study provides a comprehensive analysis of upper body kinematics during the BBT in post-stroke individuals compared to healthy people, using the IMU system. The findings underscore the feasibility and utility of IMUs for assessing motor performance in clinical settings, elucidating specific compensation strategies employed by stroke survivors.

The identification of compensation strategies, both in affected and unaffected limbs, highlights the importance of personalized rehabilitation approaches in stroke care. However, the observed overuse of the unaffected limb points out the need for interventions aimed at restoring balance between affected and unaffected limbs to optimize functional recovery. In the context of evolving rehabilitation practices, this study offers valuable insights into compensation strategies and their implications for stroke rehabilitation. Future research efforts should aim to address the limitations identified here and further elucidate the mechanisms underlying compensation strategies to inform more effective therapeutic interventions.

## Figures and Tables

**Table 1:** Demographic and clinical characteristics of the enrolled study participants.

Demographic and Clinical Characteristics (N = 62)		
	**CG (N = 31)**	**SG (N = 31)**
**Age**	62.87 ± 10.22	67.10 ± 11.06
**Gender Male,** n° (%)	16 (52%)	16 (52%)
**Arm** (n° right limb), n (%)	31 (100%)	31 (100%)
**Affected Side** (n° right limb), n (%)	-	10 (32%)
**Weight** (kg)	66.32 ± 13.99	74.38 ± 13.49
**Height** (cm)	167.12 ± 7.74	165.29 ± 9.69
**Stroke onset** (days)	-	51.30 ± 29.00
**sBBT–Dominant or Unaffected Side** (n° of cubes)	**68.06** ± 9.19 *	**52.45** ± 9.67 *
**sBBT–Non-Dominant or Affected Side** (n° of cubes)	**65.25** ± 7.35 *	**37.12** ± 12.77 *
**FMA Upper Extremity**	-	30 (11–36)
**FMA Wrist**	-	6 (0–10)
**FMA Hand**	-	9 (7–14)
**FMA Coordination/Speed**	-	4 (1–5)
**FMA TOT**	-	50 (23–65)
**FMA Sensation**	-	12 (6–12)
**FMA Passive Joint Motion**	-	24 (12–24)
**FMA Joint Pain**	-	24 (12–24)
**MAS Wrist**	-	0 (0–2)
**MAS Elbow**	-	0 (0–3)
**MAS Shoulder**	-	0 (0–3)

Abbreviations: CG, Control Group; SG, Stroke Group; sBBT, standard Box and Block Test; FMA, Fugl-Meyer Assessment; MAS, Modified Ashworth Scale. Notes: Data are reported as mean ± standard deviation or frequency (n°) with percentage (%) or median (min–max). * *p* < 0.05 (between-groups analysis).

**Table 2:** Averaged hand trajectory parameters calculated during the targeted Box and Block Test.

Hand Trajectory Parameters					
		**DA/UA**		**NDA/AA**	
		**Phase 1**	**Phase 2**	**Phase 1**	**Phase 2**
**Parameter**	**Group**	**Mean** ± **SD**	**Mean** ± **SD**	**Mean** ± **SD**	**Mean** ± **SD**
**DLJ**	CG	−3.75 ± 1.00	**−4.57** ± 2.83 *	**−3.98** ± 1.80 *	**−4.99** ± 3.96 *
	**SG**	−4.22 ± 1.28	**−5.62** ± 2.68 *	**−5.33** ± 2.11 *	**−6.45** ± 4.62 *
**LDLJ**	CG	−1.32 ± 0.01	**−1.52** ± 1.04 *	**−1.38** ± 0.58 *	**−1.61** ± 1.38 *
	**SG**	−1.44 ± 0.25	**−1.73** ± 0.98 *	**−1.67** ± 0.75 *	**−1.86** ± 1.53 *
$V_{m}(cm\;s^{-1})$	CG	**31.18** ± 5.13 *	**26.92** ± 4.56 *	**30.30** ± 4.51 *	**27.16** ± 4.42 *
	**SG**	**23.23** ± 7.29 *	**20.42** ± 6.50 *	**20.52** ± 9.46 *	**20.97** ± 13.55 *

Abbreviations: SD, standard deviation; DA/UA, Dominant Arm/Unaffected Arm; NDA/AA, Non-Dominant Arm/Affected Arm; DLJ, DimensionLess Jerk index; LDLJ, Log-DimensionLess Jerk index; $V_{m}$, mean velocity; CG, Control Group; SG, Stroke Group. Note: * *p* < 0.05 (between-groups analysis).

**Table 3:** Averaged upper limb joint angles (Phase 1).

Joint Angles						
**DA/UA**					**NDA/AA**	
**Phase 1**					**Phase 1**	
**Joint Angles**		**Group**	**Mean Angle**	**ROE**	**Mean Angle**	**ROE**
**Elbow**	F.E.	CG	**81.80** ± 21.48 *	14.87 ± 5.42	**80.95** ± 17.90 *	16.93 ± 6.24
		**SG**	**74.17** ± 18.25 *	18.85 ± 7.02	**73.04** ± 18.69 *	17.83 ± 6.70
	P.S.	CG	**71.01** ± 17.05 *	**17.85** ± 9.42 *	**68.43** ± 15.67 *	**17.66** ± 9.17 *
		**SG**	**66.35** ± 22.38 *	**28.90** ± 10.08 *	**59.59** ± 26.40 *	**25.56** ± 16.75 *
**Shoulder**	A.A.	CG	**51.19** ± 22.62 *	19.15 ± 9.54	**51.43** ± 24.05 *	20.06 ± 10.06
		**SG**	**55.25** ± 29.37 *	20.84 ± 11.20	**55.15** ± 23.80 *	20.22 ± 7.98
	F.E.	CG	42.22 ± 26.94	28.92 ± 13.28	**44.66** ± 22.99 *	**34.32** ± 12.56 *
		**SG**	43.34 ± 25.09	31.86 ± 15.59	**39.99** ± 27.38 *	**27.02** ± 12.09 *
	ROT	CG	−42.58 ± 16.20	32.48 ± 8.71	**−37.33** ± 11.95 *	**34.42** ± 8.63 *
		**SG**	−39.16 ± 15.99	30.87 ± 10.55	**−45.29** ± 26.04 *	**29.32** ± 9.41 *
**Wrist**	F.E.	CG	**19.19** ± 16.91 *	22.32 ± 13.51	**16.72** ± 17.26 *	**20.53** ± 6.74 *
		**SG**	**27.01** ± 19.52 *	23.18 ± 21.55	**19.73** ± 20.79 *	**26.44** ± 13.91 *
	URD	CG	**1.57** ± 7.62 *	8.55 ± 6.43	**−0.52** ± 9.45 *	8.55 ± 5.13
		**SG**	**4.98** ± 12.07 *	8.59 ± 10.08	**−4.06** ± 7.42 *	7.13 ± 7.06
	ROT	CG	−11.97 ± 22.95	24.85 ± 12.79	**−11.49** ± 18.23 *	22.15 ± 7.28
		**SG**	−11.05 ± 20.91	25.99 ± 21.45	**−7.18** ± 19.54 *	24.82 ± 14.77

Abbreviations: Dominant Arm (DA); Unaffected Arm (UA); Non-Dominant Arm (NDA); Affected Arm (AA); Range of Execution (ROE); flexion-extension (F.E.); Prone-Supination (P.S.); Rotation (ROT); Abduction-Adduction (A.A.); Ulnar-Radial Deviation (URD). Note: * *p* < 0.05 (between-groups analysis).

**Table 4:** Averaged trunk joint angles (Phase 1).

Joint Angles						
**DA/UA**					**NDA/AA**	
**Phase 1**					**Phase 1**	
**Joint Angles**		**Group**	**Mean Angle**	**ROE**	**Mean Angle**	**ROE**
**Pelvis**	L.B.	CG	**3.62** ± 4.58 *	3.95 ± 1.97	**4.29** ± 4.15 *	3.59 ± 1.75
		**SG**	**6.62** ± 0.57 *	3.95 ± 2.26	**6.73** ± 1.29 *	4.80 ± 2.62
	ROT	CG	4.73 ± 1.47	2.96 ± 1.20	4.77 ± 1.37	2.79 ± 1.40
		**SG**	4.69 ± 12.05	3.05 ± 1.35	4.21 ± 11.36	3.87 ± 1.82
	TILT	CG	1.39 ± 2.68	2.07 ± 1.16	**0.06** ± 3.53 *	**2.11** ± 1.53 *
		**SG**	1.98 ± 8.70	2.88 ± 2.07	**2.11** ± 7.76 *	**4.15** ± 3.02 *
**Trunk**	F.E.	CG	13.25 ± 6.39	3.24 ± 1.46	11.50 ± 7.57	**3.38** ± 1.32 *
		**SG**	11.80 ± 10.39	4.08 ± 2.02	12.25 ± 8.61	**5.73** ± 3.16 *
	L.B.	CG	**5.45** ± 5.55 *	5.18 ± 3.03	**−6.20** ± 3.13 *	6.16 ± 3.31
		**SG**	**8.74** ± 0.78 *	6.93 ± 3.21	**−9.85** ± 1.36 *	7.62 ± 3.40
	ROT	CG	**9.73** ± 2.98 *	8.39 ± 1.35	**−5.66** ± 1.52 *	7.88 ± 2.03
		**SG**	**18.79** ± 1.33 *	8.44 ± 2.48	**−20.35** ± 0.66 *	9.88 ± 2.78

Abbreviations: Dominant Arm (DA); Unaffected Arm (UA); Non-Dominant Arm (NDA); Affected Arm (AA); Range of Execution (ROE); flexion-extension (F.E.); Rotation (ROT); Lateral Bending (L.B). Note: * *p* < 0.05 (between-groups analysis).

**Table 5:** Averaged upper limb joint angles (Phase 2).

Joint Angles						
**DA/UA**					**NDA/AA**	
**Phase 2**					**Phase 2**	
**Joint Angles**		**Group**	**Mean Angle**	**ROE**	**Mean Angle**	**ROE**
**Elbow**	F.E.	CG	**84.17** ± 22.40 *	**17.50** ± 4.83 *	**85.06** ± 18.93 *	18.86 ± 7.07
		**SG**	**78.57** ± 18.23 *	**22.97** ± 7.77 *	**75.89** ± 21.36 *	21.23 ± 8.32
	P.S.	CG	61.72 ± 19.90	15.91 ± 6.63	**58.82** ± 13.51 *	18.69 ± 10.68
		**SG**	59.53 ± 24.21	19.79 ± 8.65	**51.60** ± 29.67 *	17.17 ± 6.85
**Shoulder**	A.A.	CG	53.87 ± 22.25	21.26 ± 9.51	51.64 ± 19.50	23.59 ± 10.87
		**SG**	57.82 ± 28.26	20.98 ± 10.19	53.82 ± 25.22	21.11 ± 11.18
	F.E.	CG	39.40 ± 27.36	32.18 ± 13.21	**44.38** ± 24.47 *	**35.97** ± 12.49 *
		**SG**	37.57 ± 25.80	33.50 ± 15.85	**39.33** ± 33.08 *	**31.49** ± 14.21 *
	ROT	CG	−45.54 ± 17.26	34.39 ± 8.41	**−40.77** ± 12.61 *	**37.17** ± 9.07 *
		**SG**	−44.44 ± 17.10	33.63 ± 8.91	**−49.44** ± 28.86 *	**31.58** ± 10.18 *
**Wrist**	F.E.	CG	**16.77** ± 16.73 *	26.29 ± 16.53	**13.38** ± 18.05 *	**26.29** ± 11.20 *
		**SG**	**23.14** ± 19.47 *	28.69 ± 19.22	**16.97** ± 23.00 *	**30.26** ± 13.92 *
	URD	CG	**−6.22** ± 1.65 *	7.00 ± 3.34	−10.16 ± 1.63	6.79 ± 5.11
		**SG**	**−12.41** ± 2.18 *	9.63 ± 1.31	−8.14 ± 8.78	6.98 ± 8.14
	ROT	CG	−5.90 ± 13.08	**14.27** ± 2.52 *	**−4.11** ± 7.88 *	**13.02** ± 2.42 *
		**SG**	−3.91 ± 19.82	**26.87** ± 18.01 *	**−0.76** ± 18.10 *	**26.56** ± 12.63 *

Abbreviations: Dominant Arm (DA); Unaffected Arm (UA); Non-Dominant Arm (NDA); Affected Arm (AA); Range of Execution (ROE); flexion-extension (F.E.); Prone-Supination (P.S.); Rotation (ROT); Abduction-Adduction (A.A.); Ulnar-Radial Deviation (URD). Note: * *p* < 0.05 (between-groups analysis).

**Table 6:** Averaged trunk joint angles (Phase 2).

Joint Angles						
**DA/UA**					**NDA/AA**	
**Phase 2**					**Phase 2**	
**Joint Angles**		**Group**	**Mean Angle**	**ROE**	**Mean Angle**	**ROE**
**Pelvis**	L.B.	CG	**2.75** ± 4.28 *	3.19 ± 1.89	**3.95** ± 4.06 *	2.87 ± 1.70
		**SG**	**6.73** ± 0.33 *	3.50 ± 2.31	**6.61** ± 1.68 *	4.79 ± 2.93
	ROT	CG	**5.18** ± 1.53 *	2.88 ± 1.16	**6.37** ± 1.39 *	2.61 ± 1.22
		**SG**	**12.57** ± 5.75 *	3.14 ± 1.40	**12.09** ± 4.65 *	4.08 ± 2.02
	TILT	CG	**0.44** ± 2.86 *	1.94 ± 1.13	0.24 ± 1.08	1.84 ± 1.40
		**SG**	**2.67** ± 8.79 *	2.70 ± 1.26	1.65 ± 8.13	4.09 ± 2.95
**Trunk**	F.E.	CG	12.06 ± 6.64	3.21 ± 1.34	13.01 ± 7.36	**3.25** ± 1.47 *
		**SG**	12.77 ± 10.54	4.02 ± 1.79	12.49 ± 9.06	**5.75** ± 4.26 *
	L.B.	CG	**3.87** ± 4.86 *	5.98 ± 2.99	**4.29** ± 4.46 *	5.28 ± 3.28
		**SG**	**8.36** ± 0.79 *	5.90 ± 2.52	**9.01** ± 1.28 *	7.44 ± 3.77
	ROT	CG	**4.47** ± 9.83 *	8.49 ± 2.23	**−4.95** ± 5.55 *	**8.37** ± 2.65 *
		**SG**	**8.42** ± 1.39 *	9.41 ± 3.52	**−21.50** ± 0.20 *	**11.16** ± 3.50 *

Abbreviations: Dominant Arm (DA); Unaffected Arm (UA); Non-Dominant Arm (NDA); Affected Arm (AA); Range of Execution (ROE); flexion-extension (F.E.); Rotation (ROT); Lateral Bending (L.B). Note: * *p* < 0.05 (between-groups analysis).

## Data Availability

The data associated with the paper are not publicly available but are available from the corresponding author on reasonable request.

## Abbreviations

The following abbreviations are used in this manuscript:

BBT	Box and Block Test
IMU	Inertial Measurement Unit
PD	Parkinson’s Disease
CG	Control Group
SG	Stroke Group
FMA-UL	Fugl-Meyer Assessment-Upper Limb
MAS	Modified Ashworth Scale
TACI	Total Anterior Circulation Infarct
PACI	Partial Anterior Circulation Infarct
POCI	Posterior Circulation Infarct
LACI	Lacunar Infarct
TCT	Trunk Control Test
FA	Functional Alignment
DLJ	DimensionLess Jerk index
LDLJ	Log-DimensionLess Jerk index
ROE	Range of Execution
F.E.	Flexion–Extension
P.S.	Prono–Supination
L.B.	Lateral Bending
ROT	Rotation
A.A.	Ab-/Adduction
URD	Ulnar–Radial Deviation
